# From grain to forage: A decade of mycotoxin contamination in ruminant feed in the Czech Republic (2013–2023)

**DOI:** 10.17221/74/2025-VETMED

**Published:** 2026-05-27

**Authors:** Jana Cahova, Lucie Kvasnickova, Josef Illek, Tomas Palenik, Andrea Staffa, Veronika Vlasakova, Zdenka Svobodova, Lanto Ravonjison, Martin Svoboda

**Affiliations:** ^1^Department of Animal Protection and Welfare & Veterinary Public Health, Faculty of Veterinary Hygiene and Ecology, University of Veterinary Sciences Brno, Brno, Czech Republic; ^2^Large Animal Clinical Laboratory, Faculty of Veterinary Medicine, University of Veterinary Sciences Brno, Brno, Czech Republic; ^3^Ruminant and Swine Clinic, Faculty of Veterinary Medicine, University of Veterinary Sciences Brno, Brno, Czech Republic; ^4^State Veterinary Administration, Prague, Czech Republic; ^5^State Veterinary Administration, Pardubice, Czech Republic

**Keywords:** cattle, feed, fungal toxins, occurrence pattern

## Abstract

This study is based on analytical data on mycotoxin contamination in ruminant feeds in the Czech Republic over a ten-year period (2013–2023), specifically deoxynivalenol (DON), zearalenone (ZEN), T-2 toxin (T-2/HT-2), and ochratoxin A (OTA). Analyses were performed in accredited State Veterinary Institutes using validated immunochemical and chromatographic methods. Samples included forage and concentrate feeds for dairy cows, calf feed, and fattening cattle feed. During the monitoring period, DON was detected at higher concentrations, reflecting the susceptibility of field crops to *Fusarium* infection under local environmental conditions. Forage feeds showed significantly higher concentrations of DON (472.6 ± 13.2 µg/kg), ZEN (61.7 ± 4.4 µg/kg), and T-2/HT-2 toxin (36.7 ± 2.7 µg/kg) than concentrates for dairy cows (*P* < 0.05). Additionally, ZEN levels differed significantly between concentrates for dairy cows (17.1 ± 2.3 µg/kg) and calf feed (16.2 ± 3.3 µg/kg) (*P* < 0.05). Although mycotoxin concentrations generally remained below recommended limits, a small proportion of samples exceeded guideline values for DON (0.13%) and ZEN (0.56%). These findings highlight the importance of continued monitoring and preventive strategies to manage mycotoxin risks.

Mycotoxins are toxic secondary metabolites produced by various species of filamentous fungi, primarily from genera *Aspergillus*, *Fusarium*, and *Penicillium*. These compounds are considered a significant threat to animals due to their frequent occurrence in compound feeds intended for livestock feeding (Franchino et al. 2024). In developed countries, acute mycotoxicosis in animal husbandry has become rare due to improved feed hygiene. However, subclinical mycotoxicosis remains a concern, as even low mycotoxin levels can weaken the immune system, reduce vaccine efficacy, and increase vulnerability to infections. These effects can also compromise meat quality, impacting the economic value of final products (Gott and Schwandt 2021). The most frequently monitored mycotoxins in animal feeds include aflatoxins (AFs), deoxynivalenol (DON), zearalenone (ZEN), fumonisins (FUMs), ochratoxin A (OTA), and T-2 toxin (T-2/HT-2) (Kolawole et al. 2024). Ruminants are generally less susceptible to mycotoxins than monogastric animals due to natural detoxification by rumen microbiota. However, some mycotoxins persist, and ruminants are exposed to multiple toxins from diverse feeds. Total mixed ration could be a common source of mycotoxin contamination in livestock diets and remains a significant concern due to its impact on cow health and productivity (Lecolinet and Preveraud 2021). Ruminants may also be affected by a range of mycotoxins originating from silage moulds. Identifying the specific toxin responsible for clinical symptoms is challenging, even when mycotoxins are present in the affected animal. Synergistic effects further complicate diagnosis, as toxin concentrations in tissues are often minimal (Porosnicu et al. 2024). Importantly, certain mycotoxins may even be bioactivated; for example, FUMs are not effectively degraded, ZEN can be converted to the more potent α-zearalenol, and trichothecenes may damage the gastrointestinal epithelium prior to degradation (Bandyk 2024).

Ongoing climate change, accompanied by increasingly frequent weather fluctuations (such as higher incidences of extreme weather events, floods, storms, etc.) and rising temperatures, may affect the occurrence of mycotoxins in animal feed. Milder and wetter winters alternating with warm and dry summers contribute to the development of moulds and, consequently, to the production of mycotoxins (Intergovernmental Panel on Climate Change 2023). Thus, a key projected impact of climate change is that rising temperatures will facilitate the spread, introduction, or establishment of more heat-tolerant species (Vaughan et al. 2016). Climate change has already clearly led to shifts in the occurrence of fungal species typical for specific regions. Drought and elevated temperatures have led to a shift from contamination by *Fusarium verticillioides* and FUMs to contamination by *Aspergillus flavus* and AFs. *Aspergillus* is a predominantly thermophilic fungus that was previously mainly found in sub-Saharan Africa. Due to climate change and the associated rise in temperatures in the Mediterranean region, this fungal species is becoming dominant in these areas (Fapohunda and Adewunmi 2019). Based on the results of an extensive 10-year study focused on the occurrence of mycotoxins in maize, it can be concluded that fungi of the genus *Fusarium* are currently the most prevalent, with FUMs being the dominant mycotoxins detected. However, contamination with mycotoxins produced by *Aspergillus* spp. is increasingly frequent and likely to increase in the future (Locatelli et al. 2022). In light of the OTA contamination outbreak in Italy, the study conducted by Rizzo et al. (2025) underscores the critical need for rigorous monitoring of forage quality, particularly under humid conditions conducive to fungal growth.

Prevention of mycotoxicoses in cattle is primarily based on proper feed management, regular monitoring of mycotoxin contamination, and the use of methods to decontaminate mycotoxins from feed. Implementation of preventive strategies, including the use of fungicides and improved hay storage protocols, is imperative to mitigate mycotoxin-related risks in ruminant nutrition. To minimise the risk of mycotoxicosis and related economic losses, feed manufacturers increasingly incorporate mycotoxin adsorbents as one of the preventive strategies (Machado et al. 2024; Rizzo et al. 2025). Another option is biologic methods, such as enzymatic and bioenzymatic detoxification, which have emerged as a promising, selective, and environmentally friendly approach to mycotoxin decontamination in food and feed systems. Recent reviews report that a wide range of enzymes, including oxidoreductases, hydrolases, esterases, lactonases, and peroxidases, are capable of degrading or transforming major mycotoxins such as AFs, DON, ZEN, OTA, and FUMs into less toxic metabolites (Liu et al. 2024; Zhong et al. 2025).

Among mycotoxins, only AFs and ergot alkaloids are subject to specific regulatory limits set by European legislation for animal feed. For AFs, the maximum permissible levels typically range from 5 μg/kg to 20 μg/kg, while for ergot alkaloids, the limit is generally set at 1 000 mg/kg, depending on the type of feed and animal species (European Commission 2002). Other mycotoxins, including DON, ZEN, OTA, FUMs, and T-2/HT-2 toxins, have established guidance values spanning a broader range, from 250 μg/kg to 60 000 μg/kg (European Commission 2006).

As outlined above, mycotoxin contamination of feed has significant economic consequences, including reproductive issues, increased veterinary costs, and production losses (Magnoli et al. 2019). Therefore, it is advisable to monitor mycotoxin occurrence in animal feed. Recent studies have highlighted the persistent occurrence and frequent co-contamination of multiple mycotoxins in animal feed across Europe, emphasising the need for comprehensive monitoring and regulatory measures to protect animal health. A six-year survey by Twaruzek et al. (2021) confirmed that the co-occurrence of ZEN and DON is the most common. While AFs are currently not a major concern in Poland, climate change may alter this trend. Although only 0.71% of small-grain and 0.16% of maize samples exceeded EU limits, these thresholds apply to individual mycotoxins and do not account for their combined effects. A systematic review and meta-analysis by Chhaya et al. (2024) revealed that AFs were highly prevalent in bovine feed components, being detected in 59% of samples at concentrations ranging from 2.58 μg/kg to 3.92 μg/kg. A global prevalence of 31% was reported for OTA, with levels ranging from 5.56 μg/kg to 12.41 μg/kg. Mycotoxin DON was identified in 74% of the analysed samples, with concentrations ranging from 233.17 μg/kg to 327.73 μg/kg. In the case of ZEN, the review found a 70% prevalence and concentrations ranging from 42.47 μg/kg to 66.19 μg/kg. The group of FUMs was detected in 65% of samples, with levels varying from 232.19 μg/kg to 393.07 μg/kg. Lastly, T-2 and HT-2 toxins showed a prevalence of 45%, with concentrations ranging from 23.54 μg/kg to 35.12 μg/kg. Similarly, recent monitoring data from Southern Europe confirm the widespread occurrence of mycotoxins in cattle feed. In compound feed for cattle collected in Northern Spain (Navarra) during 2019–2020 (*n* = 100), at least one mycotoxin was detected in 87% of samples. The most prevalent mycotoxins were DON, detected in 76% of samples with a mean concentration of 135.1 µg/kg; ZEN, present in 49% of samples with a mean concentration of 65.4 µg/kg; and OTA, detected in 6% of samples. Co-occurrence of two or more mycotoxins was observed in 62% of cattle feed samples, most frequently involving DON and ZEN (Munoz-Solano and Gonzalez-Penas 2023). In line with the findings, an official monitoring study conducted in Italy (2018–2022) also confirmed frequent contamination of cattle feed with *Fusarium* mycotoxins. Mycotoxins DON and ZEN were the most commonly detected toxins, with mean concentrations of 0.46 mg/kg and 0.045 mg/kg, respectively. Co-occurrence of multiple mycotoxins was frequently observed, indicating chronic low-level exposure of cattle to these compounds (Franchino et al. 2024).

## Toxicity of common mycotoxins detected in ruminant feed

The following section briefly summarises the toxicological relevance of the mycotoxins included in the present study, which were most frequently detected in feed intended for ruminants, to provide context for their potential effects on animal health.

### TRICHOTHECENES

Trichothecenes constitute a class of structurally related mycotoxins synthesised by various filamentous fungi, including species of *Fusarium*, *Myrothecium*, *Stachybotrys*, *Trichoderma*, *Trichothecium*, and *Spicellum*, which represent a significant risk to animal health (Janik et al. 2021). This study focused on some of the most toxic mycotoxins in this group, specifically T-2/HT-2 toxin and DON, which were detected in the feed samples intended for ruminants.

**T-2/HT-2 toxin**. Among trichothecenes, T-2 toxin is considered the most toxic and is metabolised to HT-2 toxin (Nayakwadi et al. 2020). Mycotoxin T-2/HT-2 primarily inhibits protein synthesis, particularly in rapidly dividing tissues, and may affect immune, hepatic, renal, nervous, and reproductive systems. Dairy cattle are generally more resistant than non-ruminants due to ruminal detoxification mechanisms, including de-epoxidation and de-acetylation (Adhikari et al. 2017). Nevertheless, exposure in cattle has been associated with reduced feed intake and milk production, gastrointestinal lesions, immunosuppression, and reproductive disorders (Janik et al. 2021; Machado et al. 2024).

**Deoxynivalenol**. Mycotoxin DON, also known as vomitoxin, is a common feed contaminant that primarily affects feed intake, growth, and immune function, particularly in monogastric animals (Murtaza et al. 2024). Ruminants show higher tolerance to DON due to ruminal microbial degradation, whereas pigs are highly sensitive and poultry display intermediate susceptibility (Chukwudi et al. 2021; Zhang et al. 2025). In cattle, chronic exposure to DON-contaminated feed has been associated with diarrhoea, soft faeces, and immunosuppression, which may contribute to reduced feed intake and performance, including decreased milk yield, although the toxin is rapidly eliminated following ingestion (Porosnicu et al. 2024). At the cellular level, DON exerts its toxic effects mainly by inhibiting protein synthesis through interaction with the 60S ribosomal subunit (Gallo et al. 2022).

### ZEARALENONE

Produced by *Fusarium graminearum* and other *Fusarium* species, ZEN is a non-steroidal mycotoxin with estrogenic activity. It is one of the three primary mycotoxins found in animal feed and is predominantly present in cereals such as maize, barley, and oats (Lv et al. 2025). Fusariotoxins such as ZEN adversely affect animal health, as their estrogenic effects cause reproductive problems (Garcia et al. 2023). Although research on cattle sensitivity to ZEN is limited, studies have reported infertility, vaginitis, vulvar enlargement, vulvo-vaginitis, decreased milk yield, and hyperestrogenism. While pigs are considered the most susceptible to ZEN, similar symptoms can occur in calves with underdeveloped rumens and in young heifers (Dogan and Dal 2022; Gnezdilova et al. 2023). Exposure to ZEN has also been shown to reduce the populations of *Lachnospiraceae* and *Prevotellaceae* in the rumen and decrease ruminal pH and total short-chain fatty acid levels, despite elevated rumination (Hartinger et al. 2022).

### OCHRATOXIN A

Mycotoxin OTA, produced by *Aspergillus* and *Penicillium* species, primarily exerts toxicity by disrupting protein synthesis and cellular transport mechanisms (Liu et al. 2022). In ruminants, OTA is partially detoxified to ochratoxin α by ruminal microbiota, resulting in lower sensitivity compared to monogastric species; however, hepatic and renal effects and low-level transfer to milk may still occur [EFSA Panel on Contaminants in the Food Chain (CONTAM) et al. 2023]. Toxic effects in ruminants are dose-dependent, with high dietary levels causing anorexia, diarrhoea, reduced milk production, and neurological symptoms, while lower doses may not elicit overt clinical signs (Kemboi et al. 2020; Rizzo et al. 2025). In addition, OTA has been shown to impair epithelial barrier integrity and modulate immune responses in bovine cells, highlighting its potential risk to animal health (Xu et al. 2022).

The aim of this study was to evaluate the occurrence of the most prevalent mycotoxins in ruminant feed in the Czech Republic over the past decade (2013–2023) based on analytical data generated by accredited laboratories and subsequently statistically evaluated. The obtained data were interpreted in relation to the recommended limit values defined by the relevant European legislation (Commission Recommendation 2006/576/EC – European Commission 2006; Commission Recommendation 2013/165/EU – European Commission 2013) to assess potential risks to ruminant health and performance.

## MATERIAL AND METHODS

Each feed sample was analysed in accordance with relevant European legislation, in particular Regulation (EC) No. 152/2009 (European Commission 2009), which outlines standardised procedures for sampling and analytical methods in the official control of feed. The samples were provided by private entities such as producers, farmers, and veterinarians, and were not collected as part of a targeted monitoring programme. As a result, sampling was driven by suspicions of mycotoxin contamination rather than random selection. Moreover, the scope of mycotoxin testing was determined by the providers, meaning that each sample was analysed only for selected groups of mycotoxins rather than a comprehensive panel. The analysis focused on detecting key mycotoxins of ruminant feed, including DON, ZEN, OTA, and T-2/HT-2 toxin. Analyses of mycotoxins were performed at the State Veterinary Institutes in Prague, Olomouc, and Jihlava using validated immunochemical and chromatographic methods. DON was primarily determined using ELISA (VERATOX kits; Neogen Corporation, Lansing, MI, USA) or liquid chromatography with diode array detection (LC-DAD), with confirmatory analysis by tandem mass spectrometry (LC-MS/MS). The analysis of ZEN was carried out using ELISA or liquid chromatography with fluorescence detection (LC-FLD), with confirmation by LC-MS/MS. FUM was determined using ELISA and confirmed by LC-MS/MS. The analysis of OTA was conducted using LC-FLD and LC-MS/MS. Over the ten-year study period, a total of 2 023 feed samples were evaluated, including calf feed (*n* = 38), concentrates for dairy cows (*n* = 766), forage for dairy cows (*n* = 1 198), and feed for fattening cattle (*n* = 21). The evaluated feed samples encompassed forages (e.g., maize silage, meadow hay, rye straw), concentrate feeds (e.g., corn cob mix, calf starter mixture, concentrates for beef cattle), as well as other complete formulated mixtures for ruminants (e.g., corn-based conserved mixture, complete dairy feed, total mixed ration for lactating cows, commercial beef finishing feed). The samples represented different animal categories, namely dairy cows, calves, and fattening cattle. The study covers a ten-year observation period from 2013 to 2023.

### Statistical analysis

The data were divided according to toxin type and feed sample. Within each group, basic descriptive statistics were calculated, including the median, mean, and the standard error of the mean. Two factors were considered in the further analysis: toxin type and feed mixture. To assess differences between groups, one factor was held constant while the other was varied. Specifically, fixing the feed mixture allowed comparisons of toxin concentrations across different toxins, whereas fixing the toxin type enabled comparisons of its concentration across different feed mixtures. Because the data did not meet the assumption of normality, nonparametric methods were applied. The significance threshold (α) was set at 0.05. Given the presence of 4 groups (4 toxins and 4 feed mixtures), the Kruskal–Wallis test was employed to evaluate overall differences. A significant result from the Kruskal–Wallis test indicated the presence of group differences, but it did not specify which groups differed. Therefore, post hoc pairwise comparisons were conducted using the Mann–Whitney test. To control for multiple testing, the Bonferroni correction was applied by multiplying each *P*-value by the number of pairwise comparisons (6).

## RESULTS AND DISCUSSION

For clarity, all statistical analyses were performed on a unified dataset covering the entire monitoring period (2013–2023). The distinction between earlier (2013–2018) and later (2019–2023) periods is used solely for interpretative purposes, reflecting changes in the spectrum of monitored mycotoxins rather than a formal division of the dataset.

### Occurrence of mycotoxins in different ruminant feeds

The mycotoxin profile summarising positive detections of individual mycotoxins in the Czech Republic from 2013 to 2023 is presented in Table 1. Across the dataset, DON was detected at elevated concentrations, consistent with its known role as a marker of *Fusarium* contamination. In calf feed, a heterogenous distribution of mycotoxin concentrations was observed (*P* < 0.05), with DON detected within higher concentration ranges. Due to the limited number of available measurements, statistical evaluation of T-2/HT-2 toxin was not possible. Across both concentrate and forage feeds intended for dairy cows, DON was consistently detected at elevated concentrations. When evaluating fattening cattle feed, DON was the most frequently detected mycotoxin. Finally, comparison between feed types revealed that forage feed for dairy cows contained significantly higher concentrations of DON, ZEN, and T-2/HT-2 toxin than concentrate feed for dairy cows (*P* < 0.05). During 2013–2023, the recommended limit for DON was exceeded in 1 out of 730 concentrate feed samples (0.13%), whereas the limit for ZEN was surpassed in 3 out of 531 forage feed samples (0.56%) according to EC Recommendation 2006/576/EC.

**Table 1:** Mycotoxin profiles across ruminant feed categories during 2013–2023 in the Czech Republic

Feed category	Mycotoxin	*n*	Mean ± SEM (µg/kg)	Median (µg/kg)	Recommended limits (EU) (µg/kg)	Samples exceeding the limit [*n *(%)]
Calf feed	DON	36	236.8 ± 49.5^a^	148.00	2 000^1^	0
	ZEN	33	16.2 ± 3.3^b^	5.00	500^1^	0
	T-2/HT-2	4	1.3 ± 1.2	0.04	250^2^	0
	OTA	18	0.4 ± 0.1^c^	0.35	250^1^	0
						
Dairy cows – concentrate	DON	730	191.0 ± 24.8^a^	3.06	5 000^1^	1 (0.13)
	ZEN	398	17.1 ± 2.3^b^	0.33	500^1^	0
	T-2/HT-2	109	12.7 ± 3.4^b^	0.03	250^2^	0
	OTA	68	1.3 ± 0.5^b^	0.10	250^1^	0
						
Dairy cows – forage	DON	1 154	472.6 ± 13.2^a^	458.50	5 000^1^	0
	ZEN	531	61.7 ± 4.4^b^	5.00	500^1^	3 (0.56)
	T-2/HT-2	630	36.7 ± 2.7^c^	5.00	250^2^	0
	OTA	9	0.6 ± 0.3^bc^	0.08	250^1^	0
						
Fattening cattle feed	DON	19	183.2 ± 97.2^a^	50.0	5 000^1^	0
	ZEN	19	11.9 ± 5.6^b^	5.00	500^1^	0
	T-2/HT-2	6	0.9 ± 0.8^c^	0.03	250^2^	0
	OTA	8	1.2 ± 1.0^bc^	0.10	250^1^	0

^a–c^Significant differences (*P* < 0.05) are indicated by different alphabetic superscripts; ^1^Commission Recommendation 2006/576/EC; ^2^Commission Recommendation 2013/165/EU

DON = deoxynivalenol*; n* = the number of positive detections for the respective mycotoxin; OTA = ochratoxin A; SEM = standard error of the mean; T-2/HT-2 = T-2/HT-2 trichothecene; ZEN = zearalenone

### Mycotoxin concentration detected in feed for ruminants in the period 2013–2023 in the Czech Republic

Analysis of DON revealed statistically significant differences (*P* < 0.05) across feed types. Calf feed contained lower DON levels than forage for dairy cows, but higher levels than dairy cow concentrates. Among dairy cow feeds, forage showed higher DON concentrations than concentrates, whereas fattening cattle feed showed lower concentrations compared with dairy cow forage. For ZEN, significant differences (*P* < 0.05) were observed between calf feed and dairy cow concentrates and between concentrates and forage for dairy cows, with higher concentrations detected in forage. Regarding T-2/HT-2 toxin, a statistically significant difference (*P* < 0.05) was identified between concentrates and forage for dairy cows. For OTA, which was monitored in the later years of the study period, no statistically significant differences (*P* > 0.05) were observed among feed types or animal categories when evaluated within the overall dataset (Table 2).

**Table 2:** Mycotoxin concentration detected in feed for ruminants in the period 2013–2023 in the Czech Republic

Mycotoxin	Feed category	*n*	Mean ± SEM (µg/kg)	Median (µg/kg)	Recommended limits (EU) (µg/kg)	Samples exceeding the limit [*n *(%)]
DON	calf feed	36	236.8 ± 49.5^b^	148.0	2 000^1^	0
	dairy cows – concentrate	730	191.0 ± 24.8^c^	3.06	5 000^1^	1 (0.13)
	dairy cows – forage	1 154	472.6 ± 13.2^a^	458.50	5 000^1^	0
	fattening cattle feed	19	183.2 ± 97.2^bc^	50.00	5 000^1^	0
						
ZEN	calf feed	33	16.2 ± 3.3^a^	5.00	500^1^	0
	dairy cows – concentrate	398	17.1 ± 2.3^b^	0.33	500^1^	0
	dairy cows – forage	531	61.7 ± 4.4^a^	5.00	500^1^	3 (0.56)
	fattening cattle feed	19	11.9 ± 5.6^ab^	5.00	500^1^	0
						
T-2/HT-2 toxin	calf feed	4	1.3 ± 1.2^ab^	0.04	250^2^	0
	dairy cows – concentrate	109	12.7 ± 3.4^b^	0.03	250^2^	0
	dairy cows – forage	630	36.7 ± 2.7^a^	5.00	250^2^	0
	fattening cattle feed	6	0.9 ± 0.8^ab^	0.03	250^2^	0
						
OTA	calf feed	18	0.4 ± 0.1^a^	0.35	250^1^	0
	dairy cows – concentrate	68	1.3 ± 0.5^a^	0.10	250^1^	0
	dairy cows – forage	9	0.6 ± 0.3^a^	0.08	250^1^	0
	fattening cattle feed	8	1.2 ± 1.0^a^	0.10	250^1^	0

^a–c^Significant differences (*P* < 0.05) are indicated by different alphabetic superscripts; ^1^Commission Recommendation 2006/576/EC; ^2^Commission Recommendation 2013/165/EU

DON = deoxynivalenol*; n* = the number of positive detections for the respective mycotoxin; OTA = ochratoxin A; SEM = standard error of the mean; T-2/HT-2 = T-2/HT-2 trichothecene; ZEN = zearalenone

The results of this study highlight the persistent presence and variability of mycotoxin contamination in ruminant feed in the Czech Republic over the past decade (2013–2023). Among the mycotoxins analysed, DON was consistently the most prevalent across all examined feed types. The higher levels of DON observed in forage feed for dairy cows, compared to other ruminant feeds, may be attributed to the greater susceptibility of field crops to *Fusarium* infection under environmental conditions typical of the region. This dominance of DON aligns with previous European studies that identified it as a widespread contaminant in cereal-based feeds and bovine feed (Twaruzek et al. 2021; Chhaya et al. 2024). Both Svoboda et al. (2019) and Mikula et al. (2020) similarly highlighted the high prevalence of DON in feedstuffs intended for pig and poultry in the Czech Republic, noting that DON concentrations occasionally exceed EU safety thresholds, posing significant risks to animal health and performance. The findings of the present study are also consistent with recent monitoring data from other European regions. Studies conducted in Southern Europe have reported widespread contamination of cattle feed with mycotoxins, with DON and ZEN being the most frequently detected contaminants, whereas OTA was detected less commonly (Munoz-Solano and Gonzalez-Penas 2023). Similarly, an official monitoring study from Italy covering the period 2018–2022 confirmed frequent contamination of cattle feed with *Fusarium* mycotoxins, particularly DON and ZEN (Franchino et al. 2024). These studies also documented the co-occurrence of multiple mycotoxins in cattle feed. Taken together, these findings support the conclusion that DON and ZEN are commonly present in cattle feed across different European regions, whereas other mycotoxins, such as OTA, tend to occur less frequently. In line with these observations, the present study indicates that contamination patterns were primarily driven by DON and ZEN, and co-occurrence of these mycotoxins has also been reported in cattle feed in Central Europe, including Poland (Twaruzek et al. 2021). In cattle, chronic exposure to DON-contaminated feed has been linked to diarrhoea, immunosuppression, and reduced feed intake and milk production (Porosnicu et al. 2024). On the other hand, ZEN may impair reproductive performance and rumen function in cattle, particularly in young animals, potentially leading to fertility disorders and reduced productivity (Dogan and Dal 2022; Gnezdilova et al. 2023). Together with the occurrence of DON and ZEN in ruminant feed, these finding raise concerns about potential health implications. Although ruminants can partially detoxify these toxins via ruminal microbiota, prolonged exposure to elevated levels can impair animal health and productivity (Lecolinet and Preveraud 2021; Garcia et al. 2023).

A change in the spectrum of detected mycotoxins was observed over the monitoring period, with OTA detected in the later years of the study. However, no statistically significant differences in OTA concentrations were observed across the analysed feed categories. These findings are likely influenced by the limited number of OTA-positive samples, which was substantially lower than for the other monitored mycotoxins, and should therefore be interpreted with caution. Mycotoxin OTA, primarily produced by *Aspergillus* and *Penicillium* species, typically occurs under warmer conditions ranging from 25 °C to 30 °C (Wang et al. 2016). In contrast, samples analysed during the earlier years contained detectable levels of T-2/HT-2 toxin, reflecting conditions favourable for *Fusarium* spp. growth under high-moisture and moderately warm conditions around 20 °C (Verheecke-Vaessen et al. 2021). The presence of OTA in the later period and T-2/HT-2 toxin in the earlier one reflects the focus of the respective analyses, which were conducted based on recommendations from sample providers. While this may indicate a possible shift in mycotoxin patterns, the conclusion remains uncertain, as it is based on a single set of targeted analyses and does not provide conclusive evidence of a replacement of T-2/HT-2 toxin by OTA.

Although OTA was detected at lower concentration levels, its occurrence in ruminant feed may represent a potential emerging risk, particularly given OTA’s known nephrotoxic and immunosuppressive effects in ruminants (Kemboi et al. 2020; Rizzo et al. 2025). While sheep, cattle, and goats can partially degrade OTA in the rumen, acute ochratoxicosis remains rare. Proper rumen function represents an important barrier limiting systemic absorption of OTA, and appropriate feeding strategies play a key role in reducing the risk of ochratoxicosis in ruminants (Rizzo et al. 2025).

For mycotoxins monitored in the later years of the study, the absence of statistically significant differences among feed types may indicate a more uniform contamination pattern, potentially reflecting improvements in feed handling or changes in agricultural practices that reduce localised contamination. Nevertheless, consistently higher mycotoxin concentrations observed in forage compared with concentrate feed across the dataset highlight the importance of monitoring individual feed ingredients separately, particularly those derived from field crops.

Overall, these findings emphasise the necessity for continued, comprehensive surveillance of multiple mycotoxins in ruminant feeds, considering their frequent co-occurrence and possible synergistic toxic effects. Monitoring programmes should integrate climatic data to anticipate and manage emerging risks associated with environmental change. Furthermore, adopting effective preventive strategies, including feed processing controls, mycotoxin binders, and improved storage conditions, remains critical to safeguarding animal health and farm productivity amid evolving mycotoxin challenges.

In conclusion, it can be stated that this study confirms the persistent occurrence of multiple mycotoxins in ruminant feed in the Czech Republic over a ten-year monitoring period (2013–2023). Across the dataset, DON was the most prevalent mycotoxin, particularly in forage feed for dairy cows, reflecting the susceptibility of field crops to *Fusarium* infection. Mycotoxin OTA was detected mainly in the later years of the monitoring period, indicating a change in the spectrum of monitored mycotoxins rather than a direct temporal shift in contamination patterns. During the monitoring period, exceedances of recommended limits were infrequent, occurring in a small proportion of samples for DON in concentrates and ZEN in forage feed. These findings highlight the importance of continued, integrated monitoring and preventive strategies to manage evolving mycotoxin risks and safeguard animal health and productivity.

## References

[R1] Adhikari M, Negi B, Kaushik N, Adhikari A, Al-Khedhairy AA, Kaushik NK, Choi EH. T-2 mycotoxin: Toxicological effects and decontamination strategies. Oncotarget. 2017 May 16;8(20):33933-52. 28430618 10.18632/oncotarget.15422 PMC5464924

[R2] Bandyk CA. Review: Mycotoxins in ruminant livestock production: An underestimated and overlooked risk and opportunity? Appl Anim Sci. 2024 Dec;40(6):802-17.

[R3] Chhaya RS, O’Brien J, Nag R, Cummins E. Prevalence and concentration of mycotoxins in bovine feed and feed components: A global systematic review and meta-analysis. Sci Total Environ. 2024 Jun 15;929:172323. 38608906 10.1016/j.scitotenv.2024.172323

[R4] Chukwudi UP, Kutu FR, Mavengahama S. Mycotoxins in maize and implications on food security: A review. Agric Rev. 2021;42(1):42-9.

[R5] Dogan V, Dal SD. Negative effects of zearalenone on reproductive productivity in dairy cattle. Vet J Kastamonu Univ. 2022 Jun;1(1):42-57.

[R6] EFSA Panel on Contaminants in the Food Chain (CONTAM); Schrenk D, Bignami M, Bodin L, Chipman JK, Del Mazo J, Grasl-Kraupp B, Hogstrand C, Hoogenboom LR, Leblanc JC, Nielsen E, Ntzani E, Sand S, Schwerdtle T, Vleminckx C, Wallace H, Gropp J, Antonissen G, Rychen G, Gomez Ruiz JA, Innocenti ML, Rovesti E, Petersen A. Risks for animal health related to the presence of ochratoxin A (OTA) in feed. EFSA J. 2023 Nov 7;21(11):e08375. 37942224 10.2903/j.efsa.2023.8375 PMC10628734

[R7] European Commission. Commission Directive 2002/32/EC of 7 May 2002 on undesirable substances in animal feed. Off J Eur Union. 2002;L 140:10-22.

[R8] European Commission. Commission Recommendation of 17 August 2006 on the presence of deoxynivalenol, zearalenone, ochratoxin A, T-2 and HT-2 toxins, and fumonisins in products intended for animal feed. Off J Eur Union. 2006;L 229:7-9.

[R9] European Commission. Commission Regulation (EC) No 152/2009 of 27 January 2009 laying down the methods of sampling and analysis for the official control of feed. Off J Eur Union. 2009;L 54:1-130.

[R10] European Commission. Commission Recommendation 2013/165/EU of 27 March 2013 on the presence of T-2 and HT-2 toxin in cereals and cereal products. Off J Eur Union. 2013;L 91:12-5.

[R11] Fapohunda SO, Adewunmi AA. Climate change and mycotoxins: The African experience. Croat J Food Sci Technol. 2019;11(2):283-90.

[R12] Franchino C, Vita V, Iammarino M, De Pace R. Monitoring of animal feed contamination by mycotoxins: Results of five years of official control by an accredited Italian laboratory. Microorganisms. 2024 Jan 15;12(1):173. 38257999 10.3390/microorganisms12010173 PMC10819248

[R13] Gallo A, Mosconi M, Trevisi E, Santos RR. Adverse effects of Fusarium toxins in ruminants: A review of in vivo and in vitro studies. Dairy. 2022 Jul;3(3):474-99.

[R14] Garcia I, Valente D, Carolino N, Dinis H, Sousa R, Duarte SC, Silva LJG, Pereira AMPT, Pena A. Occurrence of zearalenone in dairy farms – A study on the determinants of exposure and risk assessment. Toxicon. 2023 Mar 15;225:107051. 36804606 10.1016/j.toxicon.2023.107051

[R15] Gnezdilova L, Fedotov S, Muradyan Z, Rozinsky S. Effect of mycotoxins on reproduction and milk quality of dairy cattle and its amelioration by natural adaptogen “Betulin”. Qubahan Acad J. 2023 Dec;3(4):400-8.

[R16] Gott P, Schwandt E. Mycotoxins in dairy cattle: What we know and what we can do. Am Assoc Bovine Pract Conf Proc. 2021 Oct;54(2):94-101.

[R17] Hartinger T, Grabher L, Pacifico C, Angelmayr B, Faas J, Zebeli Q. Short-term exposure to the mycotoxins zearalenone or fumonisins affects rumen fermentation and microbiota, and health variables in cattle. Food Chem Toxicol. 2022 Apr;162:112900. 35247503 10.1016/j.fct.2022.112900

[R18] Intergovernmental Panel on Climate Change. Climate change 2022: Impacts, adaptation and vulnerability. Contribution of Working Group II to the Sixth Assessment Report of the Intergovernmental Panel on Climate Change. Portner HO, Roberts DC, Tignor M, Poloczanska ES, Mintenbeck K, Alegria A, Craig M, Langsdorf S, Loschke S, Moller V, Okem A, Rama B, editors. Cambridge: Cambridge University Press; 2023.

[R19] Janik E, Niemcewicz M, Podogrocki M, Ceremuga M, Stela M, Bijak M. T-2 toxin – The most toxic trichothecene mycotoxin: Metabolism, toxicity, and decontamination strategies. Molecules. 2021 Nov 14;26(22):6868. 34833960 10.3390/molecules26226868 PMC8618548

[R20] Kemboi DC, Antonissen G, Ochieng PE, Croubels S, Okoth S, Kangethe EK, Faas J, Lindahl JF, Gathumbi JK. A review of the impact of mycotoxins on dairy cattle health: Challenges for food safety and dairy production in Sub-Saharan Africa. Toxins (Basel). 2020 Apr 2;12(4):222. 32252249 10.3390/toxins12040222 PMC7232242

[R21] Kolawole O, Siri-Anusornsak W, Petchkongkaew A, Elliott C. A systematic review of global occurrence of emerging mycotoxins in crops and animal feeds, and their toxicity in livestock. Emerg Contam. 2024 Sep;10(3):100305.

[R22] Lecolinet M, Preveraud D. Mycotoxins in dairy cows: Understanding the role of the rumen [Internet]. Antony: Adisseo; 2021 [cited 2025 Jan 1]. Available from: https://www.adisseo.com/eu/technical-articles/mycotoxins-in-dairy-cows-the-role-of-rumen-scientific-article.

[R23] Liu M, Zhang X, Luan H, Zhang Y, Xu W, Feng W, Song P. Bioenzymatic detoxification of mycotoxins. Front Microbiol. 2024 Jul 18;15:1434987. 39091297 10.3389/fmicb.2024.1434987 PMC11291262

[R24] Liu WC, Pushparaj K, Meyyazhagan A, Arumugam VA, Pappuswamy M, Bhotla HK, Baskaran R, Issara U, Balasubramanian B, Mousavi Khaneghah A. Ochratoxin A as an alarming health threat for livestock and human: A review on molecular interactions, mechanism of toxicity, detection, detoxification, and dietary prophylaxis. Toxicon. 2022 Jul 15;213:59-75. 35452686 10.1016/j.toxicon.2022.04.012

[R25] Locatelli S, Scarpino V, Lanzanova C, Romano E, Reyneri A. Multi-mycotoxin long-term monitoring survey on North-Italian maize over an 11-year period (2011–2021): The co-occurrence of regulated, masked and emerging mycotoxins and fungal metabolites. Toxins (Basel). 2022 Jul 29;14(8):520. 36006184 10.3390/toxins14080520 PMC9416020

[R26] Lv Q, Xu W, Yang F, Wei W, Chen X, Zhang Z, Liu Y. Reproductive toxicity of zearalenone and its molecular mechanisms: A review. Molecules. 2025 Jan 23;30(3):505. 39942610 10.3390/molecules30030505 PMC11821083

[R27] Machado M, Nora L, Zanin TBN, Bissacotti BF, Morsch VM, Vedovatto M, Pelisser G, Mendes RE, Galvao AC, Speroni CS, Gloria EM, Rodrigues MF, Wagner R, Stefani LM, da Silva AS. Impacts of intake of trichothecenes (Fusarium sporotrichioides) for dairy calves: Effects on animal growth, oxidative and inflammatory response. Microb Pathog. 2024 May;190:106605. 38428470 10.1016/j.micpath.2024.106605

[R28] Magnoli AP, Poloni VL, Cavaglieri L. Impact of mycotoxin contamination in the animal feed industry. Curr Opin Food Sci. 2019 Oct;29:99-108.

[R29] Mikula P, Blahova J, Honzlova A, Kalinova J, Macharackova P, Rosmus J, Svobodova Z, Svoboda M. Occurrence of mycotoxins in complete poultry feeds in the Czech Republic – Multiannual survey (2013–2018). Vet Med-Czech. 2020 Nov;65(11):487-94.

[R30] Munoz-Solano B, Gonzalez-Penas E. Co-occurrence of mycotoxins in feed for cattle, pigs, poultry, and sheep in Navarra, a region of Northern Spain. Toxins (Basel). 2023 Feb 22;15(3):172. 36977063 10.3390/toxins15030172 PMC10057204

[R31] Murtaza B, Wang L, Li X, Nawaz MY, Saleemi MK, Khatoon A, Yongping X. Recalling the reported toxicity assessment of deoxynivalenol, mitigating strategies and its toxicity mechanisms: Comprehensive review. Chem Biol Interact. 2024 Jan 5;387:110799. 37967807 10.1016/j.cbi.2023.110799

[R32] Nayakwadi S, Ramu R, Kumar Sharma A, Kumar Gupta V, Rajukumar K, Kumar V, Shirahatti PS, Rashmi L, Basalingappa KM. Toxicopathological studies on the effects of T-2 mycotoxin and their interaction in juvenile goats. PLoS One. 2020 Mar 26;15(3):e0229463. 32214355 10.1371/journal.pone.0229463 PMC7098593

[R33] Porosnicu I, Ailincai LI, Neculai-Valeanu AS, Ariton AM, Mares M. Effects of mycotoxins on the health status of dairy cattle. Sci Pap Anim Sci Biotechnol. 2024 Oct;57(2):69-78.

[R34] Rizzo M, Licata P, Niutta PP, Pugliese M, Macaluso V, Costa GL, Bruschetta G, Bruno F. An unusual outbreak of ochratoxicosis associated with Trigonella foenum-graecum ingestion in ruminants from different farms of Sicily. Toxins (Basel). 2025 Mar 2;17(3):120. 40137893 10.3390/toxins17030120 PMC11946147

[R35] Svoboda M, Blahova J, Honzlova A, Kalinova J, Macharackova P, Rosmus J, Mejzlik V, Kukol P, Vlasakova V, Mikulkova K. Multiannual occurrence of mycotoxins in feed ingredients and complete feeds for pigs in the Czech Republic. Acta Vet Brno. 2019;88(3):291-301.

[R36] Twaruzek M, Skrzydlewski P, Kosicki R, Grajewski J. Mycotoxins survey in feed materials and feedingstuffs in years 2015–2020. Toxicon. 2021 Oct 30;202:27-39. 34562492 10.1016/j.toxicon.2021.09.005

[R37] Vaughan M, Backhouse D, Del Ponte EM. Climate change impacts on the ecology of Fusarium graminearum species complex and susceptibility of wheat to Fusarium head blight: A review. World Mycotoxin J. 2016 Nov;9(5):685-700.

[R38] Verheecke-Vaessen C, Garcia-Cela E, Lopez-Prieto A, Osk Jonsdottir I, Medina A, Magan N. Water and temperature relations of Fusarium langsethiae strains and modelling of growth and T-2 and HT-2 mycotoxin production on oat-based matrices. Int J Food Microbiol. 2021 Jun 16;348:109203. 33930835 10.1016/j.ijfoodmicro.2021.109203

[R39] Wang Y, Wang L, Liu F, Wang Q, Selvaraj JN, Xing F, Zhao Y, Liu Y. Ochratoxin a producing fungi, biosynthetic pathway and regulatory mechanisms. Toxins (Basel). 2016 Mar 21;8(3):83. 27007394 10.3390/toxins8030083 PMC4810228

[R40] Xu R, Shandilya UK, Yiannikouris A, Karrow NA. Ochratoxin A and citrinin differentially modulate bovine mammary epithelial cell permeability and innate immune function. Toxins (Basel). 2022 Sep 16;14(9):640. 36136578 10.3390/toxins14090640 PMC9502480

[R41] Zhang X, Chen J, Ma X, Tang X, Tan B, Liao P, Yao K, Jiang Q. Mycotoxins in feed: Hazards, toxicology, and plant extract-based remedies. Metabolites. 2025 Mar 24;15(4):219. 40278348 10.3390/metabo15040219 PMC12029259

[R42] Zhong Q, Wu Q, Xu X, Wei W. Enzymatic detoxification of major mycotoxins: Current status, challenges, and future prospective. Mycotoxin Res. 2025 Nov;41(4):559-79. 40991165 10.1007/s12550-025-00608-y

